# The Efficacy of Intradetrusor Onabotulinumtoxin A Injection for Refractory Overactive Bladder Syndrome—A Single-Center Prospective Study

**DOI:** 10.3390/jcm14124151

**Published:** 2025-06-11

**Authors:** Chie Nakai, Kosei Miwa, Yasuhide Kitagawa, Moemi Kikuchi, Sanae Namiki, Mina Kikuchi, Kota Kawase, Koji Iinuma, Yuki Tobisawa, Keita Nakane, Takuya Koie

**Affiliations:** 1 Department of Urogynecology, Japanese Red Cross Gifu Hospital, Gifu 5028511, Japan; chie.johha@gmail.com (C.N.); k-miwa@gifu-med.jrc.or.jp (K.M.); kikuchimoemi@gmail.com (M.K.); sanaen928@gmail.com (S.N.); miepon4@gmail.com (M.K.); 2 Department of Urology, Komatsu Municipal Hospital, Komatsu 9238560, Japan; kitagawa-y@hosp.komatsu.ishikawa.jp; 3 Department of Urogynecology, Sugo Clinic, Gifu 5020914, Japan; 4 Department of Urology, Gifu University Graduate School of Medicine, Gifu 5011194, Japan; kawase.kota.b5@f.gifu-u.ac.jp (K.K.); iinuma.koji.s0@f.gifu-u.ac.jp (K.I.); tobisawa.yuki.a7@f.gifu-u.ac.jp (Y.T.); nakane.keita.k2@f.gifu-u.ac.jp (K.N.)

**Keywords:** cough-associated detrusor overactivity, urgency urinary incontinence, refractory overactive bladder, intradetrusor onabotulinumtoxin A injection therapy

## Abstract

**Background/Objectives:** Intradetrusor botulinum toxin injection is a well-established third-line therapy for patients with refractory overactive bladder (OAB) and detrusor overactivity (DO). Botulinum toxin type A (BoNT-A) is most commonly used due to its prolonged therapeutic duration. We aimed to evaluate the effectiveness of intradetrusor BoNT-A injection therapy in managing refractory OAB by performing a urodynamic study (UDS). **Methods**: The patients were prospectively enrolled between February 2020 and March 2021. The patients received treatment regimens comprising behavioral modification therapy, pelvic floor muscle physiotherapy, and/or OAB medications for at least three months. The UDS procedure was carried out by a single examiner, in accordance with the International Continence Society standards for good urodynamic practice. A total of 100 units of BoNT-A was dissolved in 10 mL of saline, and 0.5 mL (5 units) was injected at 20 sites on the posterior wall of the bladder. The primary endpoint was the change in DO, which was measured using the UDS from the baseline to two months after treatment with BoNT-A. **Results**: Prior to treatment initiation, DO was observed in all the patients during the UDS. The occurrence of DO during the filling phase demonstrated a significant decrease following treatment, with DO no longer identified in 27.3% of the patients. The first sensation of bladder filling, maximum cystometric capacity, DO, and terminal DO all demonstrated significant improvement after intradetrusor BoNT-A injection, based on the UDS. The OAB symptom scores also significantly decreased after BoNT-A therapy. **Conclusions**: The present study demonstrated that intradetrusor BoNT-A injection significantly improved symptoms in patients with OAB who had been unresponsive to various treatments. This study also demonstrated the usefulness of performing a UDS before and after treatment to prove the efficacy of BoNT-A.

## 1. Introduction

The International Continence Society (ICS) defines overactive bladder (OAB) as urinary urgency, typically accompanied by urgency urinary incontinence, frequent or nocturnal micturition, and the absence of evidence of infection or other overt pathologies [1]. The overall prevalence of OAB ranges from 10.0% to 16.0% among male patients and from 12.8% to 16.9% among female patients, with an upward trend associated with age [2,3,4]. Furthermore, the global population of patients with OAB is increasing annually [2]. These symptoms have been demonstrated to be significantly associated with a negative impact on patients’ quality of life (QOL) [5,6,7]. A review of the extant literature revealed that patients diagnosed with OAB reported lower health utility scores than those diagnosed with other chronic conditions, including asthma, cancer, diabetes, heart disease, and migraine headaches [5].

The American Urological Association and the Society of Urodynamics: Female Pelvic Medicine and Urologic Reconstruction have created and revised integrated guidelines for the treatment of OAB to help clinicians diagnose and treat patients using a stepwise approach [8,9]. First-line treatment approaches focus on behavioral modifications, including bladder retraining, fluid management, pelvic floor muscle exercises, physical therapy, and urge suppression techniques [8,9]. Second-line therapeutic options encompass oral pharmacologic agents, which can be categorized into two distinct classes, namely, antimuscarinics and β3 agonists [8,9]. Antimuscarinic agents effectively reduce OAB symptoms in elderly patients, with a minimal impact on cognition and a low incidence of central nervous system (CNS) adverse effects following short-term use [10]. Nevertheless, the potential for the prolonged use of these agents, particularly the cumulative effects of their anticholinergic properties, has raised concerns regarding cognitive decline in the elderly population [11]. While the efficacy and tolerability of β3-adrenoceptor agonists in elderly patients have been demonstrated, the long-term safety of these agents in patients with uncontrolled cardiovascular disease remains unclear [12]. Third-line therapeutic modalities have been shown to offer three options: intradetrusor onabotulinumtoxin A, peripheral tibial nerve stimulation, and sacral neuromodulation [8,9].

Intradetrusor botulinum toxin injection is a well-established treatment for patients with refractory OAB or detrusor overactivity (DO), according to several randomized controlled trials [5,13,14,15,16]. Among the various forms of botulinum toxins, botulinum toxin type A (BoNT-A) is the most commonly utilized due to its prolonged therapeutic duration, with onabotulinumtoxin A (Botox^®^, Allergan, Irvine, CA, USA) being the most frequently employed globally [17,18]. The mechanism of action of botulinum toxin is believed to involve the inhibition of acetylcholine release into the bladder endothelium [17]. BoNT-A is believed to act at four distinct sites: the neuromuscular junction, autonomic ganglion, parasympathetic postganglionic ganglion, and acetylcholine-releasing sympathetic postganglionic ganglion [17]. Treating OAB with BoNT-A injections has been the subject of three large, randomized, placebo-controlled phase III clinical studies [5,15,16]. These studies showed that the group injected with 100 units of BoNT-A experienced a reduced number of episodes of urgency urinary incontinence (UUI), improved QOL, greater patient confidence, better interpersonal relationships, and reduced social embarrassment and mental stress compared with the control group [5,15,16]. Despite the existence of numerous evaluation methodologies for intradetrusor BoNT-A injection, a paucity of studies focused on efficacy assessment through urodynamic studies (UDSs) [13,19], especially in Japan, which have not been recognized until now. Therefore, the aim of this investigation was to evaluate the effectiveness of intradetrusor BoNT-A injection for managing refractory OAB by performing a UDS before and after treatment.

## 2. Materials and Methods

### 2.1. Ethical Considerations

This investigation was authorized by the Institutional Review Board of the Japanese Red Cross Gifu Hospital (approval number: I-20090901). Because this study involved prospective data collection, we obtained written informed consent from all the enrolled patients. All the procedures involving human subjects were conducted in accordance with the Helsinki Declaration of 1964 and its subsequent amendments, in addition to equivalent ethical standards.

### 2.2. Patients

The patients were prospectively enrolled between February 2020 and March 2021. In this single-center study, patients aged ≥ 40 years with idiopathic OAB and DO, confirmed with a UDS, were eligible. Furthermore, patients who underwent treatment regimens comprising behavioral modification therapy, pelvic floor muscle physiotherapy, and/or OAB medications, including antimuscarinics or β3 agonists, for a period of at least 3 months, exhibiting either an inadequate response or intolerable adverse events, were included. Patients with stress-predominant urinary incontinence (UI), a history of pelvic abnormalities or surgery on pelvic organs, diseases affecting bladder function, or urinary tract infections were excluded from the study. Patients exhibiting symptoms such as lower urinary tract obstruction, detrusor underactivity, or a residual urine volume of greater than 100 mL after voiding were also excluded from the study.

This study collected data on several metrics, including the overactive bladder symptom score (OABSS); international prostate symptom score (IPSS); international consultation on incontinence questionnaire—short form (ICIQ-SF); and a 2-day voiding diary. These data were obtained from patients enrolled in the study before BoNT-A injection and at two weeks, three months, and six months after BoNT-A injection.

### 2.3. UDS Methods and Assessment Parameters

The UDS procedure was carried out by a single examiner in accordance with ICS standards for good urodynamic practice [20,21]. The UDS was performed using the Laborie Goby™ multichannel urodynamic system (Laborie Medical Technologies, Mississauga, ON, Canada). This system utilizes a 6Fr dual-channel urethral catheter to assess intradetrusor pressure by injecting physiological saline. Additionally, an 8Fr rectal balloon catheter was employed to measure the intra-abdominal pressure. The catheter was inserted with the patient in the supine position, and the examination was performed with the patient in the sitting position to ensure zero subtraction. The bladder was infused with normal saline at a rate of 30 mL/min. Cough tests were administered at regular intervals throughout the examination to ascertain the presence of CADO. The UDS was performed before and two months after the intradetrusor BoNT-A injection.

The maximum cystometric capacity (MCC), frequency of DO, bladder capacity at first DO (mL), and detrusor pressure (cmH_2_O) were measured using the UDS. In addition, the amount of saline administered from the start of the infusion to the MCC was calculated as the compliance of the detrusor muscle; if the patient was voiding at the time of DO appearance (terminal DO), the value immediately before that time was used as the compliance of the detrusor muscle. Furthermore, the presence of DO leak point pressure, cough-related detrusor overactivity (CADO), and terminal DO were assessed. The residual urine volume was measured with catheterization before and after the UDS.

### 2.4. Intradetrusor BoNT-A Injection Therapy

Male patients underwent spinal anesthesia for intradetrusor BoNT-A injection, while female patients underwent bladder mucosal infiltration anesthesia with 60 mL of 2% lidocaine for 15 min. Levofloxacin was administered orally for a period of three days, commencing the day before the surgical procedure to prevent urinary tract infection. BoNT-A was injected using a 22-G BoNee injection needle (Coloplast, Humlebaek, Denmark) via a rigid cystoscope. A total of 100 units of BoNT-A was dissolved in 10 mL of saline, and 0.5 mL (5 units) was injected at 20 sites on the posterior wall of the bladder. The injection of BoNT-A into the triangle or dome of the urinary bladder was avoided in order to prevent the occurrence of vesicoureteral reflux or the possibility of accidental puncture into the abdominal cavity [22]. The male patients underwent a procedure in which a balloon catheter was placed and were discharged the following day. The female patients were discharged on the same day, contingent on the confirmation of the absence of hematuria.

### 2.5. Endpoints and Statistical Analyses

The primary endpoint was the change in DO, measured using the UDS, from the baseline to two months after treatment with BoNT-A. Secondary endpoints assessed the following variables two weeks after BoNT-A administration: changes from the baseline in episodes of urinary frequency, urinary symptoms, and urgency; changes from the baseline in OABSS, IPSS, and ICIQ-SF; and changes from the baseline in bladder capacity, detrusor muscle strength, and residual urine volume, as assessed by the UDS. Urinary diaries were used to monitor urinary frequency, UI, and urgency. Wilcoxon’s signed-rank test and Fisher’s exact test were used to statistically analyze the changes in various variables before and after BoNT-A administration. Statistical significance was defined as a two-sided *p*-value < 0.05.

## 3. Results

### Patient Characteristics

A total of 13 patients (nine women and four men) with refractory OAB who provided informed consent for intradetrusor BoNT-A injection were included in the study. The patients’ characteristics before BoNT-A injection are displayed in Table 1. Two patients with missing data were excluded from this analysis. The patients enrolled in the study exhibited a tendency to be on medications for relatively extended periods and use a greater quantity of drugs.

Alterations in voiding diaries and symptom score variables before and after BoNT-A intradetrusor injection are shown in Table 2. In all the patients, UI resolved within two weeks of BoNT-A intradetrusor injection, and 53.8% of the patients were able to void completely. The OABSS total score demonstrated a substantial decrease from its baseline value; however, the score exhibited an adverse change three months after treatment. The residual urine volume (RUV) escalated from 6.2 mL prior to treatment to 99.7 mL after intradetrusor BoNT-A injection, and two of the patients (11.8%) required temporary intermittent catheterization (CIC) due to urinary retention. Urinary tract infection with fever was observed in one case; however, no other adverse events were documented.

Alterations in the UDS parameters before and after BoNT-A intradetrusor injection are shown in Table 3. Prior to treatment initiation, DO was observed in all the patients during the UDS. The occurrence of DO during the filling phase demonstrated a significant decrease following treatment, with DO no longer identified in 27.3% of the patients. CADO decreased from six (54.5%) to three (27.3%) after treatment, although this decrease was not statistically significant.

## 4. Discussion

The patients in this study exhibited a longer treatment history than those in previously reported studies, with a median duration of OAB medication use prior to treatment of six years and a median number of OAB medications used of four [5,15]. Furthermore, all the patients enrolled in this study exhibited UI. Conversely, BoNT-A injection demonstrated efficacy in ameliorating not only their urinary frequency, but also their urgency and night-time frequency. Moreover, it significantly improved their OABSS and ICIQ-SF scores. The findings of this study imply that intradetrusor BoNT-A injection may be an important treatment option for patients with OAB that is refractory to various treatments.

Oral anticholinergics or β3-adrenoreceptor agonists represent the mainstay of first-line pharmacological therapies for OAB; however, many patients discontinue their use because of insufficient efficacy and/or intolerable adverse events (AEs) [23,24,25,26]. Anticholinergics are associated with a multitude of bothersome and persistent AEs, including constipation, dry mouth, falls, fractures, cognitive impairment, and dementia [23]. Its influence on the CNS was examined in a retrospective cohort study using the Taiwan National Health Insurance Research Database [26]. This cohort study included 2540 patients diagnosed with diabetes [26]. The enrolled patients were stratified into two groups: those who received oxybutynin, tolterodine, and solifenacin and those who did not receive anticholinergic drugs, and the cumulative risk of a new diagnosis of dementia during the 12-year follow-up period was examined [26]. Compared with the patients not receiving anticholinergics, the adjusted hazard ratios (HRs) for a diagnosis of dementia increased by 135% (HR, 2.35; 95% confidence interval [CI], 1.96–2.81) in patients receiving solifenacin and by 124% (HR, 2.24; 95% CI, 1.85–2.73) in those receiving tolterodine [26]. Similarly, exposure to anticholinergic OAB medications, such as oxybutynin, solifenacin, tropium, and fesoterodine, was associated with an increased risk of dementia in a nested case–control study that followed 4810 patients with dementia and 24,050 matched controls from the French Health Data Hub for five years [27]. Therefore, to avoid the risk of cognitive decline, delirium, falls, and fractures, several guidelines recommend β3-adrenergic receptor agonists as an initial therapy for OAB [8,9]. In the PILLAR study, a phase IV, double-blind, randomized trial comparing mirabegron and a placebo, the most common treatment-emergent AEs (TEAEs) in the patients treated with mirabegron were urinary tract infection, headache, and diarrhea [24]. Among the patients treated with mirabegron, the incidence rate of TEAEs increased slightly more in the patients ≥ 75 years of age than in those < 75 years of age [24]. In the Phase III EMPOWUR study, the incidence of cardiovascular-related AEs was lower in the patients treated with vibegron than in those treated with a placebo, and there was no difference based on patient age [25]. However, polypharmacy has been reported to be more prevalent in OAB patients aged ≥ 75 years than in those aged < 75 years, which can lead to long-term medication and adherence issues [28].

BoNT-A is a 150 kDa protein, and BoNT-A injection, when instilled intravesically, is theorized to act on the detrusor muscle via several distinct mechanisms [29,30]. In the motor pathway, BoNT-A inhibits the release of acetylcholine at presynaptic nerve endings located within the somatic and autonomic nervous systems, leading to the chemical denervation of target muscles [30]. Regarding its impact on sensory function, BoNT-A exerts its effects through the desensitization of afferents by selectively interrupting the release of various neurotransmitters, including adenosine triphosphate, substance P, and calcitonin gene-related peptide [31]. Additionally, BoNT-A downregulates sensory receptors, such as transient receptor potential vanilloid 1 and purinergic (P2X2, P2X3) receptors, which contribute to its overall therapeutic efficacy in the management of OAB symptoms [31]. The efficacy of intradetrusor BoNT-A injections has been demonstrated in patients with OAB who do not respond adequately to first-line pharmacological therapies, such as antimuscarinics and β3-adrenergic receptor agonists, or who experience intolerable adverse events [12]. Recent studies have also demonstrated the superior therapeutic efficacy of intradetrusor injections of BoNT-A compared to oral medications [29]. Additionally, research on bladder tissue biopsies performed before and after BONT-A injection therapy suggests that injecting BONT-A into the bladder mucosa has the effect of suppressing the inflammation and fibrosis of the bladder smooth muscle [32]. However, there is a paucity of studies on the therapeutic efficacy of BoNT-A in UDSs [13,19].

DO is a diagnostic finding of UDSs that indicates the presence of involuntary orthostatic contractions during the fluid accumulation [19]. These contractions can occur spontaneously or in response to external stimuli [19]. However, a preponderance of studies indicates that most cases exhibiting UUI do not manifest DO during a UDS [33]. This observation indicates that DO may not be a constant phenomenon during the filling phase [33]. Consequently, the accuracy of DO detection with UDSs is potentially compromised owing to the brevity of the examination [33]. Additionally, DO has been observed not only in patients diagnosed with OAB but also in healthy women, indicating that a UDS is not a prerequisite for the diagnosis of OAB or DO [13,34]. In this study, we evaluated patients before and after intradetrusor BoNT-A injection using a UDS. The rationale for this approach was that performing a UDS would facilitate a more comprehensive understanding of the pathophysiological mechanisms underlying the condition and potentially aid the development of more effective treatment strategies for patients with refractory OAB. In the preoperative evaluation, all the patients exhibited DO and associated reproducible UUI. Following injection, there were substantial improvements in multiple domains, including the MCC, bladder capacity at the initial DO, and bladder capacity at the initial UUI. Additionally, there was a significant decrease in the incidence of DO and terminal DO. This finding suggests that UDS evaluation may be a useful adjunct for assessing the therapeutic efficacy of BoNT-A injection. Although a UDS is not an examination that should necessarily be performed to confirm the efficacy and effectiveness of BoNT-A injection therapy in daily practice, it may be useful to objectively evaluate the efficacy of this treatment modality. In contrast, 72.7% of the patients in this study had terminal DO before treatment, which was significantly improved by intradetrusor BoNT-A injection. A previous study reported that oral medications for OAB were less effective in patients with terminal DO than in those with phasic DO [35]. The findings of this study indicate that BoNT-A injection may demonstrate notable effectiveness in patients with terminal DO; however, its efficacy appears to be diminished in approximately one-quarter of the patients. The therapeutic efficacy of BoNT-A therapy is reduced in patients with terminal DO, suggesting that the pathogenic mechanisms may differ between terminal and phasic DO [36]. While the underlying mechanisms remain to be fully elucidated, it has been postulated that uroepithelial dysfunction, myogenic hyperactivity, neuromuscular junction dysfunction, and CNS dysinhibition may contribute to the onset of DO [37]. While phasic DO is primarily attributed to uroepithelial dysfunction, terminal DO is associated with increased muscarinic receptor expression and a lack of CNS inhibition [38]. These findings suggest that patients with terminal DO may be resistant to intradetrusor BoNT-A injection.

It has been hypothesized that TEAEs are confined to the urinary tract. Although urinary tract infection (UTI), dysuria, urinary retention, and increased residual urine volume (RUV) have been observed, BoNT-A 100U injection is considered relatively safe [15]. However, the incidence of TEAEs remains significant, with 6% to 43% of patients experiencing CIC due to an RUV of ≥ 100 mL and 7% to 44% of patients experiencing UTIs [5,15,39,40]. In the present study, a significant increase in the RUV after treatment was also observed, with two patients (11.8%) requiring CIC due to urinary retention. Predictive factors associated with CIC use include the male sex, comorbidities, older age, the number of vaginal deliveries, a history of hysterectomy, the voiding capacity, UDS parameters, the bladder contractility index, and the bladder outlet obstruction index [41]. In the present study, one of the two patients with urinary retention was male, and the other patient did not exhibit any of the pre-treatment parameters associated with post-treatment urinary retention.

This study has certain limitations. First, the number of enrolled patients was limited, and the follow-up duration was comparatively protracted. Furthermore, the presence of a potential sex bias among the enrolled patients in the study may be a limiting factor in terms of the analysis of the outcomes. Second, given that the UDS was solely administered prior to and following BoNT-A injection, the efficacy of the UDS in terms of evaluating the long-term therapeutic effects of BoNT-A injection remains unconfirmed. Finally, we did not consider subsequent therapeutic interventions in the patients who failed to respond to intradetrusor BoNT-A injection.

## 5. Conclusions

The present study demonstrated that intradetrusor BoNT-A injection significantly improved the symptoms of patients with OAB who were unresponsive to various treatments. This study also demonstrated the usefulness of performing a UDS before and after treatment to prove the efficacy of BoNT-A injection. A multicenter study with a large number of patients is needed to prove the reproducibility of treatment evaluations using the UDS.

## Figures and Tables

**Table 1:** Patient characteristics at baseline.

Variables	
Age (years, median, IQR)	73 (68–77)
Female (number, %)	8 (72.7)
BMI (kg/m^2^, median, IQR)	
Male	30.2 (27.7–32.8)
Female	23.4 (21.6–25.2)
History of behavioral therapy (number, %)	6 (54.5)
History of anticholinergic use	
Duration (year, median, IQR)	6.0 (4.5–7.8)
Medications (*n*, median, IQR)	3.0 (2.5–3.0)

*n*, number; IQR, interquartile range; BMI, body mass index; POP, pelvic organ prolapse; UI, urinary incontinence.

**Table 2:** Changes in voiding diary and symptom score variables before and after BoNT-A intradetrusor injection.

	Before Injection	Two Weeks After Injection	*p*- Value	Three Months After Injection	*p*- Value	Six Months After Injection	*p*- Value
UI (*n*, median, IQR)	5 (2–7)	1 (0–2.5)	0.003				
Urgency (*n*, median, IQR)	3 (0–8)	1 (0–1.3)	0.030				
Daytime frequency (*n*, median, IQR)	12 (10–16)	9 (7–11)	0.045				
Night-time frequency (*n*, median, IQR)	4 (2.3–5)	3 (2–3)	0.040				
Daytime functional capacity (mL, median, IQR)	120 (103–131)	131 (82–145)	0.135				
Night-time functional capacity (mL, median, IQR)	220 (136–246)	204 (146–237)	0.235				
PVR (mL, median, IQR)	6 (0–20)	100 (0–250)	0.002	33 (10–65)	0.045		
OABSS total score (median, IQR)	10 (10–11)	4 (2–6)	0.002	8 (4–10)	0.052	8 (5–10)	0.104
IPSS total score (median, IQR)	16 (8.5–22.5)	13 (11.5–15.5)	0.328	15 (8–18)	0.104	13 (10.5–21.5)	0.580
ICIQ-SF total score (median, IQR)	15 (13–16)	11 (5–12)	0.005	12 (9–14)	0.051	9 (7–14)	0.098

*n*, number; IQR, interquartile range; UI, urinary incontinence; RUV, residual urine volume; OABSS, overactive bladder symptom score; IPSS, international prostate symptom score; ICIQ-SF, international consultation on incontinence questionnaire—short form.

**Table 3:** Changes in parameters of urodynamic study before and after BoNT-A intradetrusor injection.

	Before Injection	Injection After Two Months	*p*-Value
FS (mL, median, IQR)	54 (37–87)	111 (108–143)	0.004
MCC (mL, median, IQR)	220 (106–244)	238 (211–276)	0.035
Compliance (mL/mmH_2_O, median, IQR)	83 (49–184)	100 (57–136)	0.874
PdetQmax (mmH_2_O, median, IQR)	20 (13–25)	19 (11–21)	0.320
DO (*n*, median, IQR)	3 (2.5–4)	2 (1–2.5)	0.031
Capacity of first DO (mL, median, IQR)	180 (84–197)	227 (205–261)	0.006
First DO pressure (mmH_2_O, median, IQR)	10 (10–30)	22 (17–29)	0.342
DOLPP (mmH_2_O, median, IQR)	29 (18–36)	19 (14–24)	0.173
CADO (*n*, %)	6 (54.5)	3 (27.3)	0.371
terminal DO (*n*, %)	8 (72.7)	2 (18.2)	0.041

IQR, interquartile range; FS, first sensation of bladder filling; MCC, maximum cystometric capacity; DO, detrusor overactivity; DOLPP, detrusor overactivity leak point pressure; CADO, cough-associated detrusor overactivity.

## Data Availability

The original contributions presented in this study are included in the article. Further inquiries can be directed to the corresponding author. The data is not publicly available due to privacy reasons.

## Abbreviations

The following abbreviations are used in this manuscript:

AE	adverse event
BoNT-A	botulinum toxin type A
CI	confidence interval
CNS	central nervous system
DO	detrusor overactivity
HR	hazard ratio
ICIQ-SF	the international consultation on incontinence questionnaire—short form
ICS	The International Continence Society
IPSS	the international prostate symptom score
MCC	maximum cystometric capacity
OAB	overactive bladder
OABSS	the overactive bladder symptom score
QOL	quality of life
RUV	residual urine volume
TEAE	treatment-emergent adverse event
UDS	urodynamic study
UI	urinary incontinence
UTI	urinary tract infection
UUI	urgency urinary incontinence
